# Understanding the precursor frequencies of HIV-1 specific CD4+ T cells in seronegative donors

**DOI:** 10.1186/1742-4690-9-S2-P289

**Published:** 2012-09-13

**Authors:** SL Campion, T Brodie, A Rossetti, N Goonetilleke, F Sallusto, A McMichael

**Affiliations:** 1 University Of Oxford, Oxford, UK; 2 IRB, Bellinzona, Switzerland

## Background

HIV-1 specific T cell responses are detectable amongst a proportion of HIV-1 exposed, seronegative individuals. Previous studies from our group have demonstrated these responses are predominately mediated by CD4+ T cells and can be mapped and titrated at the peptide level. Curiously, approximately 1 in 4 HIV-1 un-exposed seronegative donors also have demonstrable HIV-1 specific T cell responses. This observation raises a number of questions regarding the ontogeny of pre-existing HIV-1 specific T cells and their potential role in the acquisition of HIV-1.

## Methods

A highly sensitive T cell library method was used to screen the naïve, central and effector memory CD4+ T cell subsets from 10 healthy, HIV-1 seronegative, leukapharesis donors. 192 cell lines per subset were screened against pools of overlapping 18mer peptides, spanning the entire HIV-1 proteome and proliferative responses quantified using tritiated thymidine incorporation.

## Results

HIV-1 specific CD4+ T cell response were detectable within the CD4+ T cell memory compartments of all 10 subjects tested, albeit at low frequency. HIV-1 specific CD4+ T cell responses spanned the entire HIV-1 proteome and were typically of low avidity. There was considerable variability between donors both in the proteins recognized and precursor frequencies of HIV-1 specific T cell responses. However, across all subsets tested CD4+ T cells specific for HIV-1 envelope appeared to exist at the highest precursor frequency, with Pol seemingly the least frequently targeted.

## Conclusion

We show HIV-1 specific CD4+ T cells to be detectable within the memory compartment of all 10 donors tested. In the absence of known prior exposure to HIV-1 these observations are indicative of low level cross reactivity within the immune system. The use of the T cell library technique to interrogate the naïve and memory precursor frequencies of HIV-1 specific T cells should prove beneficial in the design of novel therapeutic vaccines.

